# Genetic and Transcriptional Analysis of Human Host Response to Healthy Gut Microbiota

**DOI:** 10.1128/mSystems.00067-16

**Published:** 2016-08-30

**Authors:** Allison L. Richards, Michael B. Burns, Adnan Alazizi, Luis B. Barreiro, Roger Pique-Regi, Ran Blekhman, Francesca Luca

**Affiliations:** a Center for Molecular Medicine and Genetics, Wayne State University, Detroit, Michigan, USA; b Department of Genetics, Cell Biology, and Development, The University of Minnesota, Minneapolis, Minnesota, USA; c Department of Ecology, Evolution, and Behavior, The University of Minnesota, Minneapolis, Minnesota, USA; d Department of Pediatrics, Sainte-Justine Hospital Research Centre, University of Montréal, Montreal, Québec, Canada; e Department of Obstetrics and Gynecology, Wayne State University, Detroit, Michigan, USA; Argonne National Laboratory

**Keywords:** complex traits, gene expression, genetics, host response, host-microbiota interaction

## Abstract

Many studies have demonstrated the importance of the gut microbiota in healthy and disease states. However, establishing the causality of host-microbiota interactions in humans is still challenging. Here, we describe a novel experimental system to define the transcriptional response induced by the microbiota for human cells and to shed light on the molecular mechanisms underlying host-gut microbiota interactions. In primary human colonic epithelial cells, we identified over 6,000 genes whose expression changed at various time points following coculturing with the gut microbiota of a healthy individual. Among the differentially expressed genes we found a 1.8-fold enrichment of genes associated with diseases that have been previously linked to the microbiome, such as obesity and colorectal cancer. In addition, our experimental system allowed us to identify 87 host single nucleotide polymorphisms (SNPs) that show allele-specific expression in 69 genes. Furthermore, for 12 SNPs in 12 different genes, allele-specific expression is conditional on the exposure to the microbiota. Of these 12 genes, 8 have been associated with diseases linked to the gut microbiota, specifically colorectal cancer, obesity, and type 2 diabetes. Our study demonstrates a scalable approach to study host-gut microbiota interactions and can be used to identify putative mechanisms for the interplay between host genetics and the microbiota in health and disease.

**IMPORTANCE** The study of host-microbiota interactions in humans is largely limited to identifying associations between microbial communities and host phenotypes. While these studies have generated important insights on the links between the microbiota and human disease, the assessment of cause-and-effect relationships has been challenging. Although this relationship can be studied in germfree mice, this system is costly, and it is difficult to accurately account for the effects of host genotypic variation and environmental effects seen in humans. Here, we have developed a novel approach to directly investigate the transcriptional changes induced by live microbial communities on human colonic epithelial cells and how these changes are modulated by host genotype. This method is easily scalable to large numbers of host genetic backgrounds and diverse microbiota and can be utilized to elucidate the mechanisms of host-microbiota interactions. Future extensions may also include colonic organoid cultures.

## Abstract (precis)

The study of host-microbiota interactions in humans is largely limited to identifying associations between microbial communities and host phenotypes. While these studies have generated important insights on the links between the microbiota and human disease, the assessment of cause-and-effect relationships has been challenging. Although this relationship can be studied in germfree mice, this system is costly, and it is difficult to accurately account for the effects of host genotypic variation and environmental effects seen in humans. Here, we have developed a novel approach to directly investigate the transcriptional changes induced by live microbial communities on human colonic epithelial cells and how these changes are modulated by host genotype. This method is easily scalable to large numbers of host genetic backgrounds and diverse microbiota and can be utilized to elucidate the mechanisms of host-microbiota interactions. Future extensions may also include colonic organoid cultures.

## INTRODUCTION

A healthy, human adult contains over 1,000 species of bacteria in the gut (1). These bacteria live in a symbiotic relationship with us and compose the gut microbiota. Recent studies suggest that the gut microbiota may play a role in both physiological and pathological states. The composition of the gut microbiota has been correlated with complex diseases, such as Crohn’s disease and diabetes (2–5). The two most abundant phyla in the human gut are *Bacteroidetes* and *Firmicutes* (1). In obese individuals, the ratio of these two phyla is altered (6–8). Turnbaugh et al. showed that transplanting the fecal microbiota of an obese mouse to a germfree mouse caused greater weight gain in the recipient than in recipients that received the microbiota of lean mice (9). Goodrich et al. showed that this relationship exists even when the microbiota from obese humans is transplanted into mice (10). The microbiota has also been linked to colorectal cancer (11, 12) and to diseases not directly related to the gut, such as arthritis, Parkinson’s disease, and other types of cancer (13–16).

While there are many species that are common among humans, studies have shown that microbiome composition can vary widely across individuals (17, 18). These differences have been correlated to several factors, such as breastfeeding, sex, and diet (19–24). In addition to environmental factors, recent studies also support a key role for host genetics in shaping the gut microbiota. Indeed, microbiome composition is more similar in related individuals than in unrelated individuals (10, 25–28). One caveat of these studies is that, especially in humans, related individuals often share environments and follow similar eating habits, which have a strong effect on the microbiota. In an effort to control for this factor, other studies have attempted to estimate the role of host genetics on the microbiota in mice, where the environment can be regulated, or in groups of people that all share the same environment regardless of relatedness (29–32).

To further examine the effect of host genetic variation on the gut microbiota, some groups have performed association studies between host genotypes and microbiome composition (32–35). For example, Blekhman et al. studied 93 individuals and identified loci that are associated with microbiome composition in 15 body sites that were sequenced as part of the Human Microbiome Project (18, 33). Among loci that are associated with changes in abundance of microbiota species, Blekhman et al. found enrichment of single nucleotide polymorphisms (SNPs) that were previously identified as expression quantitative trait loci (eQTLs) across multiple tissues in the Genotype-Tissue Expression (GTEx) project (36). Additionally, microbiome composition has been found to be tissue specific and, therefore, likely influenced by host gene expression patterns in the specific tissue that interacts with the microbiota (18, 33). Together, these results suggest that host genetic variants affect microbiota composition through influencing host gene and protein expression. However, we know little about the interplay between human genetic variation, gene expression, variations in microbiota composition, and the effects of these factors on susceptibility to complex disease.

Molecular studies of genetic effects on cellular phenotypes (eQTLs, DNase I sensitivity QTLs [dsQTL], and transcription factors binding QTL mapping studies) have been successful in elucidating the link between genetic variation and gene regulation and have identified hundreds of variants associated with gene expression and transcription factor binding changes (37–42). Here, we present a novel approach to study the interaction between the microbiota, human genetic variation, and gene expression in a dynamic and scalable system. We cocultured primary human colonocytes (epithelial cells of the colon) with the gut microbiota of a healthy individual (extracted from a fecal sample) to study host cell gene expression responses to microbiota exposure. We identified over 6,000 genes that significantly change their expression in the host following microbiota exposure. These genes are enriched for genome-wide association study (GWAS) signals, suggesting that regulation of their expression is a potential mechanism for the associations found between host disease status and microbiota composition. In addition, to learn about host genetic variants that play a role in host-microbiota interactions, we studied allele-specific expression (ASE) and identified 12 genes that demonstrated an interaction between genotype and microbiota exposure. Future studies can use this approach to characterize the host response to the microbiota and determine the causal relationships in the context of specific diseases and traits.

## RESULTS

### Study design.

**FIG 1:**
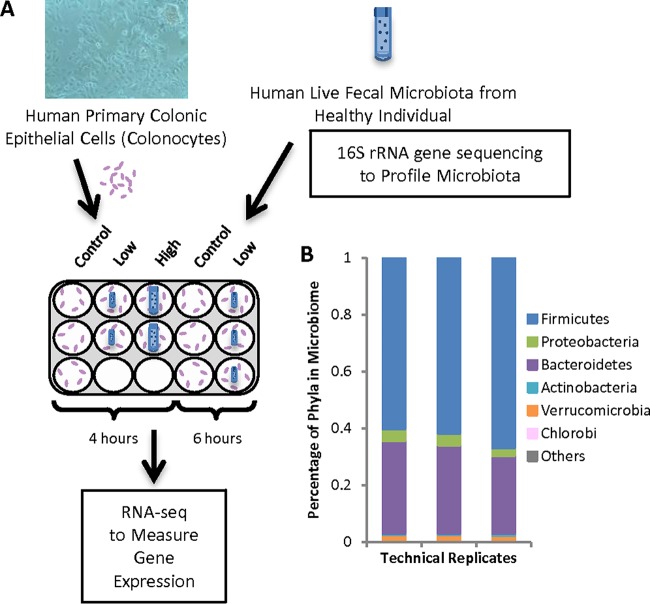
Coculturing of human colonocytes and fecal microbiota. (A) Treatment scheme to coculture colonocytes and microbiota, which was then followed by RNA sequencing of mRNA to assess host gene expression. Cells were treated for 4 and 6 h using a high or low concentration of fecal microbiota (or no fecal microbiota, as controls). (B) 16S rRNA gene sequencing results from fecal microbiota of a healthy, 22-year-old male used to coculture with colonocytes. Each bar denotes a replicate of the same uncultured fecal microbiota. The most abundant phyla are depicted as their percentages of the total microbiome detected.

While many recent studies have shown the importance of the microbiota in physiological and pathological states, in humans the direct impact of exposure to the microbiota on host cells is still unclear. To analyze the host transcriptional changes induced by a normal gut microbiota, we designed an experiment in which we cocultured human colonic epithelial cells (colonocytes) with an extract containing the fecal microbiota (Fig. 1A). The fecal microbiota was purchased from OpenBiome and was collected from a healthy individual, as characterized by a broad range of biomarkers (http://www.openbiome.org). We analyzed the DNA of the fecal microbiota through 16S rRNA gene sequencing followed by data processing using QIIME (12, 43, 44) to quantify the microbial species present (Fig. 1B; see also Table S1 in the supplemental material). To assess the technical variability introduced at any point of the microbiome analysis protocol, we sequenced three technical replicates (aliquots of the same fecal microbiota sample), and we found only minor variations. This fecal microbiome showed a normal composition of bacteria phyla, with *Firmicutes* and *Bacteroidetes* representing the most abundant taxa, consistent with previous studies of gut microbiota composition in healthy individuals (see Table S1) (18).

10.1128/mSystems.00067-16.1 Table S1  16S rRNA gene sequencing analysis of microbiome composition. Three technical replicates (aliquots of the same fecal microbiota sample) were sequenced (labeled as replicates 1, 2, and 3). The table shows proportional data from the samples rarefied to 280,000 reads. Download Table S1, XLSX file, 0.1 MB. Copyright © 2016 Richards et al. 2016 Richards et al. This content is distributed under the terms of the Creative Commons Attribution 4.0 International license.

We exposed the colonocytes to two different densities of live microbiota (measured by the optical density at 600 nm [OD_600_]), including 10:1 and 100:1 bacteria:colonocyte ratios, termed the high and low concentrations, respectively. We cultured the colonocytes in low oxygen (5% O_2_), to recapitulate the gut environment, for 4 and 6 h under three conditions: with high and low concentrations of bacteria and alone (as controls) (Fig. 1A). We collected samples after 4 and 6 h of coculturing, because culture times between 2 and 8 h have proven to be informative when determining early gene expression changes following a variety of different treatments, including infection (45–48). We also cocultured the colonocytes with 2 concentrations of microbiota, low and high, to find an optimal concentration that allowed both the colonocytes and microbial cells to thrive. The high concentration at 6 h led to reduced colonocyte viability and so it was removed, resulting in 5 experimental conditions: Low-4, Low-6, High-4, CO4, and CO6. Experimental replicates were collected for each condition: two replicates for Low-4 and High-4 and three replicates for Low-6, CO4, and CO6. We collected and sequenced the RNA in order to learn about the host cell response through study of gene expression levels and to identify genes with allele-specific expression induced by the microbiota.

### Transcriptional changes induced by the gut microbiota.

**FIG 2:**
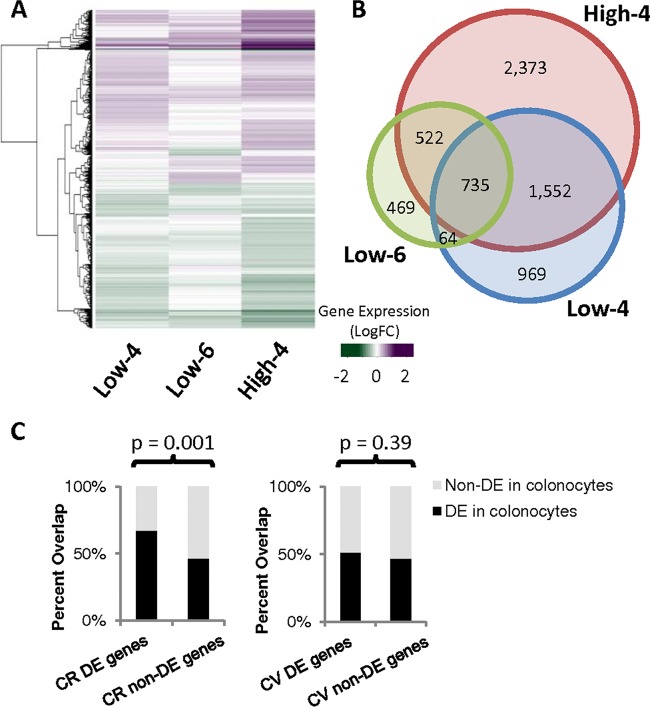
Host gene expression changes following exposure to the microbiota. (A) Heat map depicting averages across replicates of the log_2_(fold change) for each sample compared to the respective control (Low-4 and High-4 were compared to CO4, while Low-6 was compared to CO6). Green indicates a decrease in expression in the treatment sample, while purple indicates an increase in expression. One transcript from each of 6,684 genes that were DE in any of the 3 treatment groups (Benjamini-Hochberg adjusted *P* value of <0.1; |log_2_(fold change)| > 0.25) is shown. (B) Venn diagram depicting the number of genes that contained any transcript differentially expressed under the various treatment conditions. The overlap numbers require that the same gene was DE in the different samples. (C) Depiction of the percentage of DE genes from Camp et al. (either in CR or CV mice) that were DE in colonocytes in our study. *P* values are from a Fisher exact test on a two-by-two contingency table, looking at the overlap of DE genes in our data compared to DE genes in either CR or CV mice.

First, we searched for genes that were differentially expressed (DE) in the colonocytes following exposure to the gut microbiota. We used DESeq2 (49), as described in Materials and Methods to characterize differential gene expression in the treatment samples, across replicates. We focused on genes with significant differences by using a Benjamini-Hochberg adjusted *P* value of <0.1 and |log_2_(fold change)| of >0.25. For each treatment, we compared the differences in expression to the those in time-matched controls. This was done to account for any differences in expression that were independent of the treatment but may depend on other factors, such as cell cycle and the extended time under the specific culturing condition (antibiotic-free medium and 5% oxygen). With this method, we identified 3,320 genes that changed expression in Low-4 relative to CO4 (55% upregulated), 1,790 genes in Low-6 relative to CO6 (57% upregulated), and 5,182 genes in High-4 relative to CO4 (49% upregulated), resulting in 6,684 genes that had at least one transcript that was DE under any of the three conditions (Fig. 2; see also Fig. S1A and Table S2 in the supplemental material). When we compared DE genes across treatment conditions, we found that over 50% of DE genes overlapped, with 735 genes that were DE under all 3 treatment conditions (Fig. 2B). Additionally, the heat map for genes DE in any of the 3 treatment groups (Fig. 2A) showed that even genes that may not reach the significance threshold in a given treatment still changed similarly across all treatments compared to their respective controls. More specifically, while High-4 seemed to have the same gene expression changes as Low-4 but to a greater extent, Low-6 was the least concordant sample. This can also be seen by principal-component analysis (PCA), where the first PC separated the 5 samples by time (see Fig. S1B), suggesting that longer culturing times may have a global effect on gene expression, possibly affecting host cell viability.

10.1128/mSystems.00067-16.2 Table S2  Differentially expressed genes in colonocytes following coculturing. Low-4 and High-4 were compared to CO4, while Low-6 was compared to CO6, to determine gene expression changes; changes are shown for each transcript, with the gene IDs and symbols in the last 2 columns. Download Table S2, XLSX file, 3.6 MB. Copyright © 2016 Richards et al. 2016 Richards et al. This content is distributed under the terms of the Creative Commons Attribution 4.0 International license.

10.1128/mSystems.00067-16.9 Figure S1  Gene expression changes following coculturing with microbiota. (A) Each plot depicts gene expression changes for a different treatment group, compared to the time-specific control sample. The *x* axis shows the log_2_(fold change) (FC), and the *y* axis shows the −log_10_(Benjamini-Hochberg adjusted *P* value). The points colored light blue are significantly differentially expressed [Benjamini-Hochberg adjusted *P* value, <0.1, |log_2_(fold change)| > 0.25]. (B) PCA of expression across all 5 samples. (C) Enrichment of immune-related GO categories of genes that were significantly increased in expression in High-4 compared to Low-4. Download Figure S1, JPG file, 0.2 MB. Copyright © 2016 Richards et al. 2016 Richards et al. This content is distributed under the terms of the Creative Commons Attribution 4.0 International license.

To determine whether our results recapitulated gene expression patterns observed in *in vivo* models, we performed a comparison to an existing data set and assessed the effect of the microbiota on colonic gene expression in mice (50). Camp et al. studied mice that were divided into three groups: conventionally raised (CR), mice raised in a germfree environment and were then conventionalized with microbiota for 2 weeks (CV), and mice only raised in a germfree environment (GF) (50). Those authors performed transcriptome sequencing (RNA-seq) and identified 194 and 205 genes that were differentially expressed in colonic epithelial cells in CR and CV mice, respectively, compared to GF mice. When we searched for the overlap of the 6,684 DE genes in our data set, we found a significant enrichment for genes differentially expressed in CR mice (42 genes out of 63 DE genes in CR mice with orthologs in humans; Fisher’s exact test, *P* = 0.001, odds ratio [OR], 2.3) but not with CV mice (45 genes out of 88 genes with human orthologs DE in CV mice; Fisher’s exact test *P* = 0.39) (Fig. 2C). These findings suggest that our model more accurately represents a normal, healthy interaction with the microbiota, compared to the acute response observed in the CV mice.

**FIG 3:**
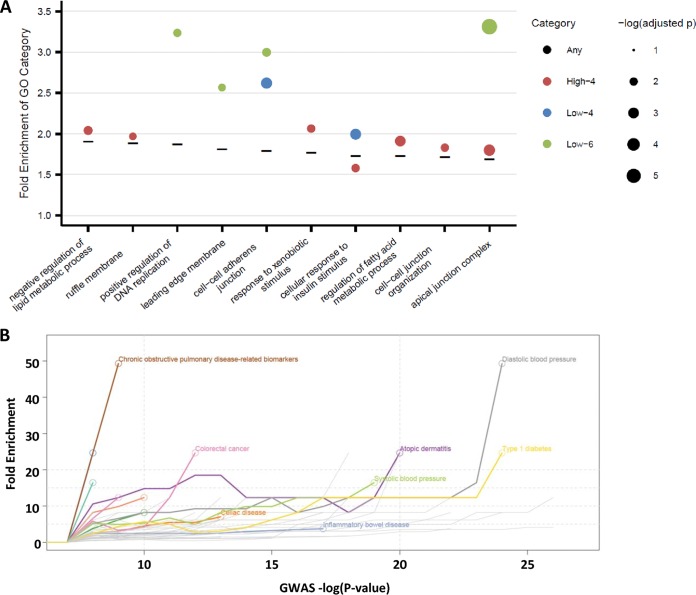
Functional enrichment of differentially expressed genes. (A) GO enrichment was assessed using GeneTrail (95) for any gene differentially expressed in any of the 3 treatment groups (6,684 genes). Enrichments for the top 10 categories that were overrepresented are indicated with a black dash (details in Materials and Methods). GO enrichment was performed for genes differentially expressed in each of the 3 treatment groups separately, and if these categories were significantly overrepresented, the enrichment in that category is shown by a closed circle (Low-4 is blue, High-4 is red, Low-6 is green). The closed circles were weighted based on the −log_10_(Benjamini-Hochberg adjusted *P* value). The black dash indicates enrichment among genes that were differentially expressed under any of the 3 treatment conditions. (B) Fold enrichment of DE genes (*y* axis) among genes associated in GWAS for a given disease at progressively stringent *P* value thresholds (*x* axis). For each GWAS and *P* value cutoff, we identified the overlap between the genes significantly associated with the disease at that cutoff and DE genes in our study, and we calculated a fold enrichment (plotted along the *y* axis), defined as the ratio of observed/expected overlap between the two gene sets. Colored lines indicate an enrichment significant at *P* < 0.05 (using Fisher’s exact test), with the point of maximum enrichment indicated by a circle. The GWAS disease name is listed next to the line for diseases with a fold enrichment of >30 or *x* axis position with maximum enrichment of >10.

We next examined the function of the genes that had changed expression levels in the host. We identified genes involved in pathways previously shown to be affected by exposure to microbiota, including cell-cell junctions (51, 52) and lipid metabolism (50, 53) (Fig. 3A). Similar to Camp et al. (50), we also identified changes in gene expression of transcription factors. Specifically, Camp et al. identified 75 differentially expressed transcription factors with binding sites enriched near genes differentially expressed between CV and CR mice. We found that in our analysis, 50 out of the 75 transcription factors were differentially expressed, corresponding to 2.3-fold enrichment over non-differentially expressed genes (Fisher exact test *P* = 0.0004) (see Table S3 in the supplemental material). These transcription factors included *EGR1*, a gene involved in the intestinal response to injury (54), and several *STAT* genes, which are part of a pathway that is upregulated in colorectal cancer (55). This overlap suggests that our *in vitro* system accurately depicted an *in vivo* response and that the changes in host gene expression are mediated by changes in the abundance of key transcription factors in humans, as Camp et al. had seen in mice.

10.1128/mSystems.00067-16.3 Table S3  Transcription factor overlap with Camp et al. binding sites. The motif name is shown as reported for binding sites in mice in the Camp et al. study. Download Table S3, XLSX file, 0.01 MB. Copyright © 2016 Richards et al. 2016 Richards et al. This content is distributed under the terms of the Creative Commons Attribution 4.0 International license.

Previous reports from animal models have demonstrated an enrichment for genes involved in the immune response among those that change expression following short-term and long-term exposure to the microbiota (50, 56, 57). Indeed, we found similar GO categories, such as “immune system process” (GO:0002376), were enriched (Benjamini-Hochberg adjusted *P* < 0.05) among genes that are differentially expressed following coculturing. While we identified immune-related categories among genes differentially expressed among all three conditions, we wondered whether immune response activation is stronger under certain conditions. Specifically, we hypothesized that colonocytes cocultured with a high dose of microbiota for 4 h would have a stronger immune response than colonocytes cocultured with a low dose for 4 h. We identified 2,094 genes that were DE between the High-4 and Low-4 groups. We found that transcripts from 1,308 genes showed increased expression at the higher concentration and transcripts from 788 genes showed decreased expression at the higher concentration of microbiota (see Table S4 in the supplemental material). When we searched among the genes that were increased in expression with the higher concentration of microbiota, we found several immune-related GO categories (see Fig. S1C in the supplemental material). These data suggested that a higher microbiota concentration elicits a stronger immune response in host cells.

10.1128/mSystems.00067-16.4 Table S4  Differences in gene expression between High-4 and Low-4. Changes in expression are shown for each transcript. Download Table S4, XLSX file, 0.5 MB. Copyright © 2016 Richards et al. 2016 Richards et al. This content is distributed under the terms of the Creative Commons Attribution 4.0 International license.

### Transcriptional response and human diseases.

The impact of microbiota exposure on gene expression led us to ask whether these changes affect human diseases. Several diseases have been linked to variation in the composition of the gut microbiome, including obesity, type 2 diabetes, inflammatory bowel disease, Crohn’s disease, ulcerative colitis, and colon cancer (7, 27, 58–65). Many GWAS analyses have identified genetic loci associated with these diseases (66), but in most cases, the mechanism by which the gene influences the disease is still unclear. Similarly, the mechanisms by which microbiota composition may influence human diseases are mostly still unknown. Our data allowed us to investigate these questions, using primary human colonic cells.

First, we hypothesized that if we identified a differentially expressed gene in our data that was also associated with a disease, it was likely that changing the expression of this gene is a mechanism by which the microbiota can affect host health. To test this hypothesis, we studied genes that were previously reported to be associated with any complex trait (NHGRI GWAS database) (66), as defined in Materials and Methods. We searched among genes that were differentially expressed, in the same direction, in all 3 treatment groups, and we found enrichment for genes associated with complex traits (Fisher’s exact test *P* < 10^−10^; OR, 1.8). We then focused on several diseases that have already been linked to microbiome composition. We found that DE genes were enriched for genes associated with obesity-related traits (Fisher’s exact test *P* = 0.03; OR = 1.5) and colorectal cancer (Fisher’s exact test *P* = 0.01; OR, 3.0) with suggestive enrichment for inflammatory bowel disease (Fisher’s exact test *P* = 0.06; OR, 1.7) and ulcerative colitis (Fisher’s exact test *P* = 0.09; OR, 1.9). There was no significant enrichment for type 2 diabetes or Crohn’s disease (see Table S5 in the supplemental material). Additionally, we found that the enrichment of genes associated with colorectal cancer was also significant when we used a complementary approach that accounted for the differences in the distribution of *P* values across GWAS (Fig. 3C). For this analysis, we used a range of −log_10_(*P* value) cutoffs for each disease in the GWAS catalog, and we identified the overlap between the genes significantly associated with the disease at each cutoff and DE genes in the current study. Using this approach, we also found enrichment among several autoimmune diseases that have been previously linked to variation in the microbiome, such as atopic dermatitis, celiac disease, and inflammatory bowel disease (67–69). These results support our system as a useful method for studying the genes through which the microbiota may interact with the host and affect human complex traits. Moreover, dysregulation of the genes that were both differentially expressed and associated with these diseases may represent a mechanism that causes the pathological state through the host cell response to the gut microbiota. Future studies utilizing microbiota from healthy and diseased individuals will be able to shed further light on how different microbes may influence disease risk through changes in host gene expression.

10.1128/mSystems.00067-16.5 Table S5  Enrichment of GWAS traits among DE genes. Six traits that have previously been linked to the microbiota were assessed. A two-by-two contingency table was constructed with the following categories: “ALL” (genes that contain transcripts that are differentially expressed, in the same direction, in all three treatment groups) or “NOT” (any other gene expressed in this study); “TRAIT” (genes that are associated with the shown trait in the GWAS catalog) or “OTHER GWAS” (genes associated with another trait in the GWAS catalog). A Fisher’s exact test was performed to obtain the *P* values and odds ratios. The last column shows genes that are DE, in the same direction, in all three treatment groups, and are found in GWAS traits related to the microbiome. These genes were used to make “ALL/TRAIT” categories. A gene may be listed more than once if it is associated with more than one trait. Download Table S5, XLSX file, 0.01 MB. Copyright © 2016 Richards et al. 2016 Richards et al. This content is distributed under the terms of the Creative Commons Attribution 4.0 International license.

### Allele-specific expression.

Genetic variants associated with microbiome composition have previously been linked to expression changes in humans through eQTL studies (33). However, to date, there are no reports for humans on the effects of genetic variants on the host transcriptional response to the microbiota. In order to identify genetic loci that may influence host-gut microbiota interactions through their influence on gene expression, we studied ASE (37–42). This analysis is ideal for our study (using colonocytes from a single individual), as it uses the genotypes and allelic imbalance for each individual separately to assess genetic control, as opposed to using multiple individuals to determine a correlation in a population between genotypes and expression (37–42). The caveat is that we can only assess SNPs that are heterozygous in our sample and deeply covered by sequencing reads. To characterize ASE in our samples, we utilized QuASAR (70), a method to detect heterozygous sites in a sample and utilize these sites to identify ASE. We found an average of 5,984 heterozygous SNPs per sample covered by at least 20 RNA-seq reads. Among these heterozygous sites, we identified 131 events of ASE at 87 SNPs in 69 unique genes (Storey false-discovery rate [FDR], <10%) across our samples, including controls (see Fig. S2A and B and Tables S6 and S7 in the supplemental material). Three of these SNPs showed the same ASE in all samples, suggesting that these may play a role in the baseline regulation of colonocytes. Forty ASE events (at 30 SNPs) occurred in the treatment samples, and 18 events (at 16 SNPs) occurred in genes that were differentially expressed at the same time point (see Table S6). This suggests that these ASE events may be a result of either new transcription of the favored allele or specific degradation of the other allele. The 22 remaining ASE events may involve genes where there are changes in expression of transcripts containing both alleles such that the gene expression remains constant though the ASE may change. We observed a difference in the proportion of ASE between the two controls, which could be due to incomplete power and technical variation. The lower proportion of ASE detected after 6 h (in both the control and the cocultured sample) may also reflect changes in regulation of colonocyte gene expression after prolonged culturing in low oxygen. These data reinforce the validity of comparing each cocultured sample to a control cultured under the same conditions (without microbiota exposure) for the same length of time. Additionally, the differences in ASE across samples may also be caused by differences in sequencing depth. While the total number of reads for each sample was similar, they each had various numbers of heterozygous sites that had sufficient coverage to test for ASE.

10.1128/mSystems.00067-16.6 Table S6  Allele-specific expression in colonocytes. The ASE is shown for each SNP in the treatment or control sample in which ASE was found. Positive β indicates higher expression for the reference allele. The last 3 columns indicate which SNPs (1) have ASE in all samples including controls, (2) have ASE in at least 1 treatment sample, or (3) are found in a gene DE at the same time point for which we identified ASE. Download Table S6, XLSX file, 0.02 MB. Copyright © 2016 Richards et al. 2016 Richards et al. This content is distributed under the terms of the Creative Commons Attribution 4.0 International license.

10.1128/mSystems.00067-16.10 Figure S2  Allele-specific expression and cASE results following coculturing with microbiota. (A) QQ-plot depicting the ASE nominal *P* values for heterozygous SNPs in human colonocytes. (B) Percentage of SNPs with allele-specific expression in each of the 5 samples (3 treatments, 2 controls), normalized by the number of heterozygous sites covered by at least 20 reads. (C) Examples of cASE following exposure to the microbiota. Forest plots depict cASE for 7 SNPs. ASE is shown for samples where at least 20 reads cover the indicated SNP (positive β indicates ASE favoring the reference allele). Download Figure S2, JPG file, 0.2 MB. Copyright © 2016 Richards et al. 2016 Richards et al. This content is distributed under the terms of the Creative Commons Attribution 4.0 International license.

**FIG 4:**
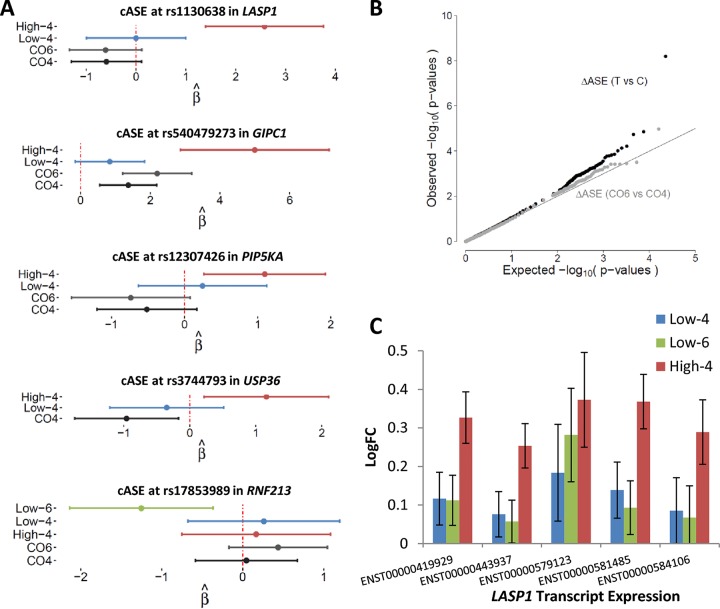
Gene-by-environment interactions in human colonocytes. (A) Forest plots depicting conditional cASE for 5 randomly chosen SNPs from the 12 with cASE (remaining are shown in Fig. S2C in the supplemental material). Allele-specific expression is shown for samples with at least 20 reads covering the indicated SNP (samples without 20 reads are not shown, as we could not accurately measure ASE). Positive β indicates allele-specific expression favoring the reference allele. (B) QQ-plot showing the nominal *P* values of SNPs that could be tested for cASE (20 reads covering SNP in both a treatment and the corresponding control or for both CO6 and CO4). (C) Gene expression changes in each treatment group (compared to the corresponding control) for each of 5 transcripts of *LASP1* expressed in colonocytes. Error bars indicate standard errors for log_2_(fold change) among replicates.

We then formally tested whether host transcriptional response may be modulated by an interaction between host genetics and the microbiota. Previous studies have examined gene-by-environment interactions in response to infection by searching for response expression quantitative trait loci (reQTLs), where the genetic effect on gene expression is only present under certain conditions (71–74). However, this type of study requires many individuals in order to gain enough statistical power. Instead, we searched for gene-by-environment interactions by examining ASE conditional on the exposure to the microbiota (conditional ASE [cASE]). Due to differences in sequencing depth and number of ASE identified in each sample, we only tested for cASE on SNPs with sufficient coverage in both the treatment and the corresponding control. We identified 12 SNPs in 12 different genes that showed cASE under any of the three treatment conditions (empirical FDR, <12%) (Fig. 4A and B; see also Table S8 and Fig. S2C in the supplemental material). These genes represent the host response that is regulated by both host genetics and the interaction with the gut microbiota.

Two of the 12 genes with cASE have been implicated in the immune response (*USP36*, *PIP5K1A*), while 8 of them have been linked to a disease affected by dysbiosis in the gut (*USP36*, *PIP5K1A*, *AFAP1L2*, *GIPC1*, *ASAP2*, *RNF213*, *KCTD12*, *LASP1*) (75–85). For example, we found cASE at SNP rs1130638, favoring the reference allele, in *LASP1* as well as increased total expression of *LASP1* following exposure to the high concentration of microbiota at 4 h (Fig. 4A and C). This suggests that the gut microbiota has a stronger effect on *LASP1* upregulation in the presence of a specific allelic variant. Because previous reports have shown that *LASP1* has increased expression in colorectal cancer (80, 81, 83), these results suggest that microbiota exposure may increase the risk for colorectal cancer in individuals carrying the allele associated with higher expression of *LASP1*.

## DISCUSSION

The gut microbiota has been shown to be complex and variable under physiological and pathological conditions. While studies of the microbiome have become more common, in humans they have been mostly limited to identifying associations between microbial communities and host phenotypes. Here, we have developed a novel approach to directly investigate the transcriptional changes induced by live microbial communities on host colonic epithelial cells and how these changes are modulated by host genotype. The advantage of this method, compared to *in vivo* studies in mice, is that it allows for high-throughput testing of multiple microbiota and host combinations with quick assessment of the interaction. Future applications of this method may extend to colonic organoids. Organoid cultures closely resemble the three-dimensional structure of the colonic epithelium observed *in vivo* and can be derived from individual biopsy specimens (86).

In this study, we were able to learn about the human colonocyte response to fecal microbial communities. We identified over 6,000 host genes that change expression following coculture with the microbiota. These genes are enriched for certain functions, including cell-cell interaction and cell migration, and in higher concentrations of microbiota we saw enrichment for genes involved in the immune response. Our study design allowed us to assess how the host-microbiota relationship changes under various conditions (Low-4, Low-6, and High-4). Our results suggest that a higher microbiota concentration leads to a more pronounced immune response that may be more representative of an infection. We also observed that prolonged coculturing (6 h) results in the least concordant gene expression changes, suggesting that culturing for 6 h may lead to lower cell viability (as could also be seen following visual inspection of the host cells). Therefore, while there is a high degree of overlap of differentially expressed genes across all 3 treatments, our data suggest that a 10:1 ratio for 4 h is the optimal treatment condition, as the genes that change expression are those involved in cell-cell junctions and cell viability is still high.

Although we did not assess changes to the composition of the microbiota following culturing, the composition may change due to the molecular interactions between host cells and microbiota, similar to changes that occur *in vivo*. However, the composition of the microbiota could also be influenced by the culturing environment. For example, the nutritional environment and oxygen level may impact the microbiota. Moreover, even though we cultured in antibiotic-free medium, residual antibiotics may still affect community composition. Future studies that profile microbiota dynamics in the culturing system will be able to address these questions.

In addition to our novel experimental design, our analysis also adds to the understanding of the interaction between human genetic variation and the microbiota. Previous work has searched for quantitative trait loci that are associated with the abundance of certain bacteria, but these studies have lacked power to detect many loci (32, 33, 35). Our analysis of allele-specific expression maximized the information available for each individual and allowed us to identify 12 loci that demonstrated conditional allele-specific expression and evidence of gene-by-microbiota interaction in a single individual. This system is easily amenable to scaling up in order to perform eQTL and response eQTL analysis (37–42, 71–74).

When we further searched for genes where genetic variation affected the response to microbiota exposure, we found 12 genes containing cASE. Several of these genes can be linked to cell adhesion and migration (*AFAP1L2*, *PIP5K1A*, *GIPC1*, *ARFGAP3*, *ASAP2*, *LASP1*) (84, 85, 87). These interactions demonstrate how the microbiota may influence cell-cell junctions and cell surface receptors, likely due to the *in vivo* reaction of colonocytes to protect the body from infection by sealing tight junctions and replacing cells that have been sloughed off by intestinal movement (52, 88–90). This influence may have an adverse effect on the host, as demonstrated by cASE for *LASP1*. *LASP1* encodes a protein that binds to actin and regulates the cytoskeleton, and it has previously been shown to increase in expression following infection. Specifically, infection with hepatitis B virus X increased *LASP1* expression and led to cell migration (97). However, when *LASP1* expression was knocked down following exposure to the virus, subsequent cell migration and movement were also reduced. Furthermore, colorectal cancer cells also show higher expression of *LASP1*, suggesting that *LASP1* plays a similar role in colonocytes (80, 81, 83). Together, these data suggest another mechanism by which the microbiota may influence cell migration and perhaps carcinogenesis through genotype-dependent expression changes in *LASP1*.

Additionally, we identified several genes with cASE that have been associated with diabetes (*GIPC1*, *USP36*, *RNF213*, *KCTD12*) or obesity (*PIP5K1A*) (78, 79, 81–83). Both diabetes and obesity have been linked to microbiome composition (7, 27, 58). These genes may play a role in host-microbiota interactions and the dysbiosis that leads to these diseases.

Our study demonstrates a scalable approach to study host-gut microbiota interactions that depicts the *in vivo* relationship. This technique allowed us to start deciphering the impact of the microbiota on host cells and will help to determine how the microbiota may lead to disease through its influence on host cell gene regulation. We also highlight the importance of gene-by-microbiota interactions and suggest that it is not simply the genetics of an individual but the interplay between genetics and microbiota that can influence health and disease. Future studies using this approach with multiple individuals and microbiota will identify key host factors and microbial communities that jointly influence human disease.

## MATERIALS AND METHODS

### Cell culture and treatment.

Experiments were conducted using primary human colonic epithelial cells (HCoEpiC, lot 9810), which we also termed colonocytes (ScienCell 2950). The cells were cultured on plates or flasks coated with poly-l-lysine (PLL), according to the supplier’s specifications (ScienCell 0413). Colonocytes were cultured in colonic epithelial cell medium supplemented with colonic epithelial cell growth supplement and penicillin-streptomycin according to the manufacturer’s protocol (ScienCell 2951) at 37°C with 5% CO_2_. At 24 h before treatment, cells were changed to antibiotic-free medium and moved to an incubator at 37°C, 5% CO_2_, and 5% O_2_.

Fecal microbiota was purchased from OpenBiome and arrived frozen on dry ice. The following briefly describes the protocol by which OpenBiome processes stool samples. The sample is collected and given to a technician within 1 h. The mass of the sample is measured and transferred to a sterile biosafety cabinet. The stool sample is put into a sterile filter bag, and a sterile filtered dilutant of 12.5% glycerol is added with a normal saline buffer (0.90% [wt/vol] NaCl in water). The sample solution is then introduced to a homogenizer blender for 60 s and aliquoted into sterile bottles. The bottles are then immediately frozen at −80°C. Any sample not fully processed within 2 h of passage is destroyed.

Upon arrival in our lab, the extract was not thawed until the day of treatment. Fecal microbiota was collected from a healthy, 22-year-old male (Unit ID 02-028-C). Prior to treatment, the fecal microbiota was thawed at 30°C, and the microbial density (OD_600_) was assessed via a spectrophotometer (Bio-Rad SmartSpec 3000). Medium was removed from the colonocytes and fresh antibiotic-free medium was added to the cells, with a final microbial ratio of 10:1 or 100:1 microbe:colonocyte in each well (low and high conditions, respectively). Additional wells containing only colonocytes were also cultured in the same 24-well plate for use as controls.

Following 4 or 6 h, the wells were scraped on ice, pelleted, and washed with cold phosphate-buffered saline (PBS) and then resuspended in lysis buffer (Dynabeads mRNA Direct kit) and stored at −80°C until extraction of colonocyte RNA. Treatments of control and Low-6 groups were done in triplicate, while the Low-4 and High-4 treatments were done in duplicate. The colonocytes exposed to the high concentration of microbiota for 6 h were unhealthy, and RNA could not be collected.

### RNA library preparation from colonocytes.

Polyadenylated mRNAs were isolated from thawed cell lysates by using the Dynabeads mRNA Direct kit (Ambion) and following the manufacturer’s instructions. RNA-seq libraries were prepared using a protocol modified from the NEBNext Ultradirectional (NEB) library preparation protocol to use bar codes from BIOOScientific added by ligation, as described in reference 91. The individual libraries were quantified using the KAPA real-time PCR system, following the manufacturer’s instructions and using a custom-made series of standards obtained from serial dilutions of the phiX DNA supplied (Illumina). The libraries were then pooled and sequenced on two lanes of the Illumina Next-seq 500 in the Luca/Pique laboratory by using the high-output kits for 75 cycles and 300 cycles to obtain paired-end reads for an average of 150 million and 50 million total reads per sample, respectively.

### 16S rRNA gene sequencing and analysis of the microbiome.

Microbial DNA was extracted from the uncultured microbiota sample in triplicate by using the PowerSoil kit from MoBio Laboratories as directed, with a few modifications. Briefly, the fecal microbiota was spun, the pellet was then resuspended in 200 µl of phenol:chloroform and added to the 750-µl bead solution from the PowerSoil kit. The kit protocol was then followed, and the column was eluted in 60 µl. This eluate was then purified using the MinElute PCR purification kit (Qiagen) according to the manufacturer’s instructions.

16S rRNA gene amplification and sequencing were performed at the University of Minnesota Genomics Center (UMGC), as described by Burns et al. (12). Briefly, DNA isolated from the fecal microbiota was quantified by quantitative PCR (qPCR), and the V5-V6 regions of the 16S rRNA gene were PCR amplified. Nextera indexing primers were added in the first PCR, using the V5F primer, 5′-AATGATACGGCGACCACCGAGATCTACAC[i5]TCGTCGGCAGCGTC-3′, and V6R, 5′-CAAGCAGAAGACGGCATACGAGAT[i7]GTCTCGTGGGCTCGG-3′, where [i5] and [i7] refer to the index sequences used by Illumina. This PCR was carried out using the KAPA HiFidelity Hot Start polymerase (Kapa Biosystems) for 20 cycles. The amplicons were then diluted 1:100 and used as input for a second PCR using different combinations of forward and reverse indexing primers for another 10 cycles. The pooled, size-selected product was diluted to 8 pM, spiked with 15% phiX, and loaded onto an Illumina MiSeq instrument to generate the 16S rRNA gene sequences (v3 kit; paired-end 2 × 300 bp), resulting in 2.2 million raw reads per sample, on average. Bar codes were removed from the sample reads by UMGC, and the Nextera adaptors were trimmed using CutAdapt 1.8.1.

The trimmed 16S rRNA gene sequence pairs were quality filtered (*q*-score of >20, using QIIME 1.8.0), resulting in 1.41, 1.06, and 1.53 million high-quality reads for sample replicates 1, 2, and 3, respectively (43, 44). Operational taxonomic units (OTUs) were picked using the closed reference algorithm against the Greengenes database (August 2013 release) (12, 43, 44, 92). The resulting OTU table was analyzed to determine microbial community diversity, using QIIME scripts and rarefying to 280,000 reads.

### RNA sequencing and differential gene expression analysis.

Reads were aligned to the hg19 human reference genome by using STAR (93) (https://github.com/alexdobin/STAR/releases, version STAR_2.4.0h1) and the Ensemble reference transcriptome (version 75) with the following options: STAR --runThreadN 12 --genomeDir <genome>; --readFilesIn <fastqs.gz> --readFilesCommand zcat; --outFileNamePrefix <stem> --outSAMtype BAM Unsorted; --genomeLoad LoadAndKeep, where <genome> represents the location of the genome and index files, <fastqs.gz> represents that sample’s fastq files, and <stem> represents the file name stem of that sample. For each sample, we merged sequencing replicates from the 2 different sequencing runs by using samtools (version 2.25.0). We further required a quality score of 10 to remove reads mapping to multiple locations. We used the WASP suite of tools (98) (https://github.com/bmvdgeijn/WASP; downloaded 9/15/15) for allele-specific mapping and to remove duplicates, to ensure that there was no mapping bias at SNPs. The resulting alignments were used for the following analyses, and the read counts can be found in Table S7 in the supplemental material.

10.1128/mSystems.00067-16.7 Table S7  Allele-specific expression as a function of sequencing depth. Download Table S7, XLSX file, 0.01 MB. Copyright © 2016 Richards et al. 2016 Richards et al. This content is distributed under the terms of the Creative Commons Attribution 4.0 International license.

To identify DE genes, we used DESeq2 (49) (R version 3.2.1; DESeq2 version 1.8.1) over experimental replicates for each treatment condition. DESeq2 was performed over each transcript expressed in all samples. A transcript was differentially expressed when the log_2_(fold change) was greater than 0.25 and had a Benjamini-Hochberg adjusted *P* value of <0.1 (94). A gene was considered DE if at least one of its transcripts was DE.

### Gene ontology analysis.

We utilized GeneTrail (95) to find enrichment of gene ontology terms. We compiled a list of unique genes that changed gene expression under any of the 3 conditions (Low-4, High-4, and Low-6) and determined which GO categories were under/overrepresented compared to a list of all genes expressed in colonocytes (15,781 genes). We considered a category over/underrepresented if the Benjamini-Hochberg adjusted *P* value was <0.05. Figure 3A depicts the top 10 categories overrepresented that had an expected number of genes between 10 and 500. Enrichment was calculated by dividing the observed number of genes in a category by the expected number based on the total gene set.

### Comparison of differentially expressed genes to those reported by Camp et al.

The genes from Table S3 in the report by Camp et al. (46) were mapped to their orthologs in humans via the Ensembl BioMart tool (96) tool for comparison to the genes in our data set.

### Enrichment of DE genes among genome-wide association studies.

We downloaded the GWAS catalog (version 1.0.1) (66) on 5 January 2016. To identify the overlap between DE genes in our data set and those associated with a GWAS trait, we intersected genes that contained transcripts that changed significantly and in the same direction in all 3 treatment groups with the reported genes from the GWAS catalog. We report enrichment with specific categories from the GWAS catalog: “Obesity-related traits,” “Inflammatory bowel disease,” “Ulcerative colitis,” “Colorectal cancer,” “Type 2 diabetes,” and “Crohn’s disease.” We used Fisher’s exact test and a two-by-two contingency table by using 2 groups: genes that contained transcripts that were DE, in the same direction, in the 3 treatment groups (“ALL”), and other genes that were expressed in each sample (“NOT”). We then split these groups into two subgroups: genes that were associated with the select disease (“TRAIT”) and genes that were associated with any other trait in the GWAS catalog (“OTHER GWAS”). Values are shown in Table S5 in the supplemental material.

### Joint genotyping and ASE inference.

First, we identified SNPs to be studied for ASE. We used all 1KG SNPs from the phase 3 release (v5b.20130502; downloaded on 8/24/15) but removed SNPs if their minor allele frequency was less than 5% or they were found in annotated regions of copy number variation and ENCODE-blacklisted regions (39). The resulting 7,340,521 SNPs were then studied in the following analysis.

Using samtools mpileup and the hg19 human reference genome, we obtained the read counts at each SNP in each sample from the RNA-seq data. These pileups were then processed using the QuASAR package (70) by combining the RNA-seq reads from each sample (as they are all derived from the same colonocyte cell line) for joint genotyping. From the genotype information we identified heterozygous SNPs with read coverage of at least 20 and we tested them for ASE by using QuASAR (70). Because all samples were collected from the same host cell line, we used read counts combined across all samples and all replicates to call genotypes. However, when we study ASE, we study each sample separately, only combining the 75- and 300-cycle runs across the experimental replicates.

### Analysis of cASE.

To identify cASE, we transformed the Quasar β parameters to differential *Z* scores (*Z*_Δ_) by using the following formula: *Z*_Δ_ = (β_T_ − β_C_)/√(se_T_^2^ + se_C_^2^), where β and se represent the estimates for the ASE parameter and its standard error (se) for either the treatment (T) or control (C) samples.

The *Z*_Δ_ scores were then normalized by the standard deviation across *Z*_Δ_ scores corresponding to control versus control (controls at 4 and 6 h). Finally, *P* values (*P*_Δ_) were calculated from the *Z*_Δ_ scores as follows: *P*_Δ_ = 2 × pnorm(−|z|). Under the null hypothesis, *Z*_Δ_ values are asymptotically normally distributed. To further correct for this small deviation, we used the control-versus-control *P* values to empirically estimate the FDR. A list of significant cASE SNPs (empirical FDR, <12%) is provided in Table S8 in the supplemental material.

10.1128/mSystems.00067-16.8 Table S8  Conditional allele-specific expression following exposure to microbiota. ASE is shown for the treatment in which cASE occurs and its respective control. dASE values indicate the significant difference between ASE in the treatment and control. Download Table S8, XLSX file, 0.01 MB. Copyright © 2016 Richards et al. 2016 Richards et al. This content is distributed under the terms of the Creative Commons Attribution 4.0 International license.

### Accession number(s).

All 16S rRNA gene sequencing data of uncultured microbiota and RNA sequencing data of colonocytes under all conditions were submitted to the Sequence Read Archive (SRA) under accession number SRP080110.
