# Supplementary figures and images for: Genetic and Transcriptional Analysis of Human Host Response to Healthy Gut Microbiota
Source: mSystems. 2016 Aug 30;1(4):e00067-16. doi: 10.1128/mSystems.00067-16 (PMC5047527; doi:10.1128/mSystems.00067-16)

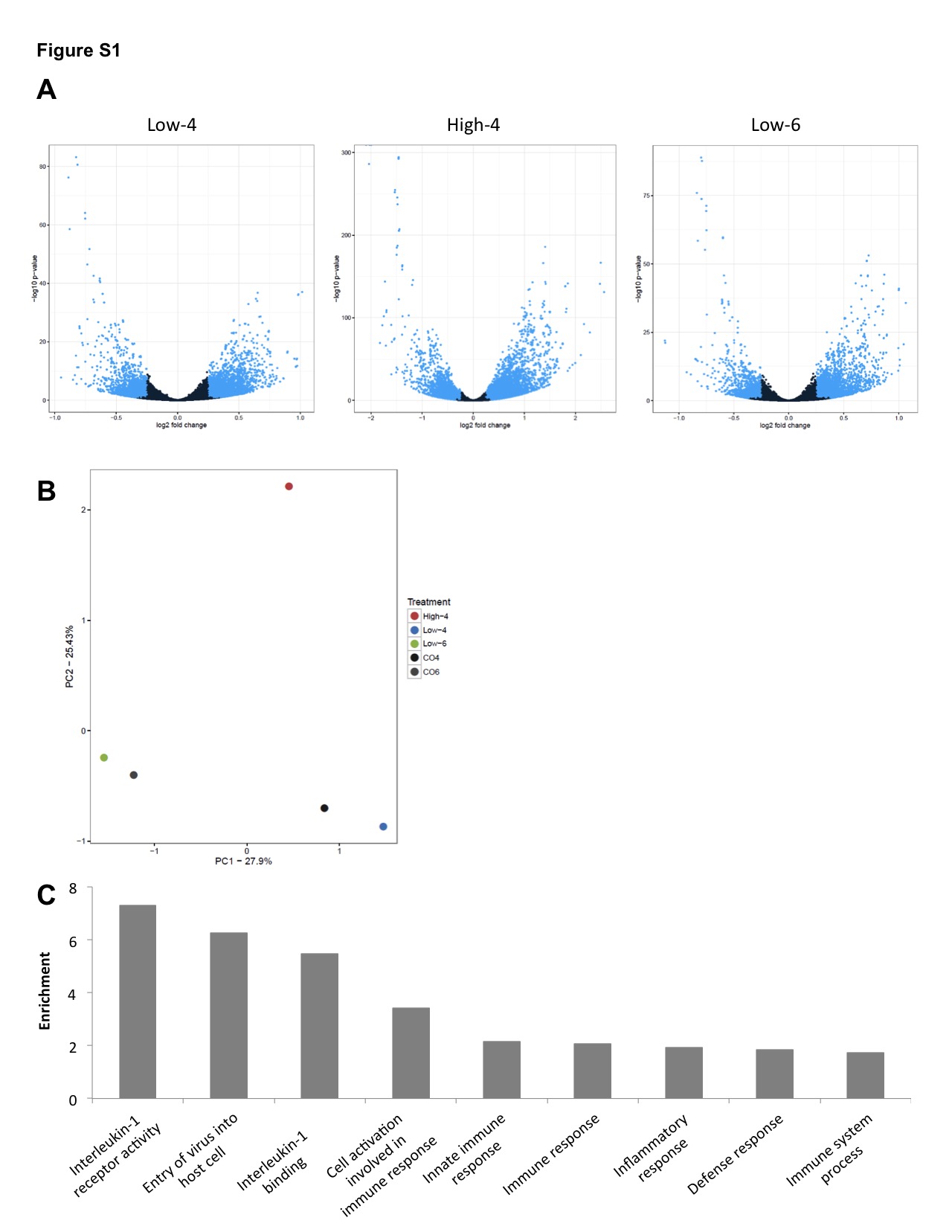

Supplement: Figure S1 [file sys004162046sf1.jpg]

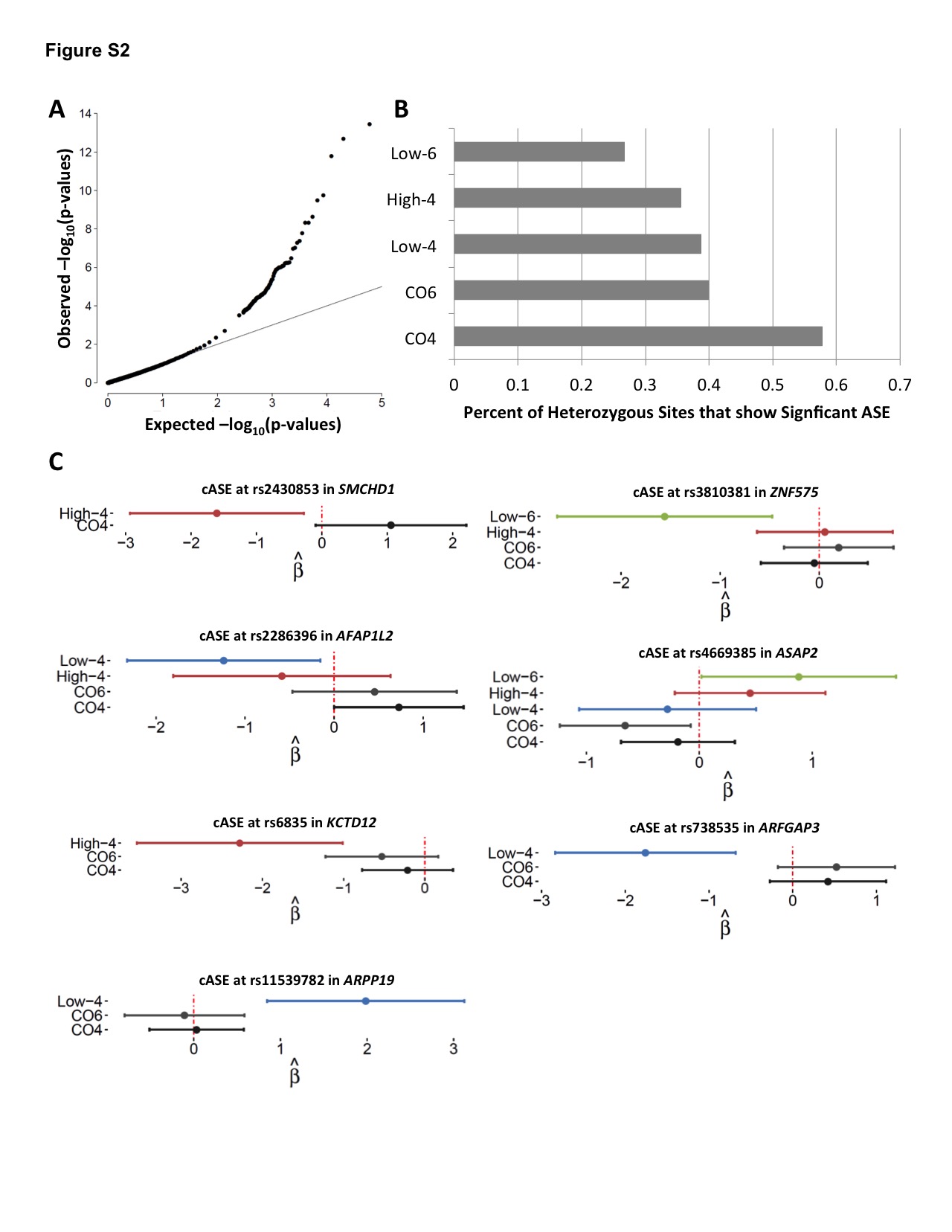

Supplement: Figure S2 [file sys004162046sf2.jpg]
